# Development and Standardization of Indirect ELISA for African Swine Fever Virus Using Recombinant p30 Protein Produced in Prokaryotic System

**DOI:** 10.3390/vetsci12100995

**Published:** 2025-10-15

**Authors:** José Luis Cerriteño-Sánchez, José Bryan García-Cambrón, Perla Lucero Zavala-Ocampo, Llilianne Ganges, Julieta Sandra Cuevas-Romero

**Affiliations:** 1 Centro Nacional de Investigación Disciplinaria en Salud Animal e Inocuidad, Instituto Nacional de Investigaciones Forestales, Agrícolas y Pecuarias, Cuajimalpa de Morelos, Mexico City 05110, Mexico; cerriteno.jose@inifap.gob.mx; 2 Biología Experimental, Universidad Autónoma Metropolitana Iztapalapa, Mexico City 09340, Mexico; tlcbioexp@gmail.com; 3 Ciencias de la Producción y de la Salud Animal, Facultad de Medicina Veterinaria, Universidad Nacional Autónoma de México, Av. Universidad 3004, Copilco Universidad, Mexico City 04510, Mexico; plucerozavala@gmail.com; 4 Instituto de Investigación y Tecnología Agroalimentarias (IRTA), Animal Health, Centre de Recerca en Sanitat Animal (CReSA), Campus de la Universitat Autònoma de Barcelona (UAB), 08193 Bellaterra, Spain; llilianne.ganges@irta.cat; 5 Unitat Mixta d’investigació IRTA-UAB en Sanitat Animal, Centre de Recerca en Sanitat Animal (CReSA), Campus de la Universitat Autònoma de Barcelona (UAB), 08193 Bellaterra, Spain

**Keywords:** p30, African swine fever, synthetic gene, recombinant protein, serovigilance

## Abstract

**Simple Summary:**

African Swine Fever (ASF) is a viral disease in pigs with major impacts on production and the economy. The development of highly sensitive and specific detection tools would enable early identification. Since the p30 protein is highly conserved in the virus, the objective of this study was to produce the p30 protein in a prokaryotic system and develop a highly sensitive and specific indirect ELISA to identify antibodies against ASFV. Our results indicate a good production yield and a good immunogenic response in a murine model. The developed ELISA showed high sensitivity and specificity with a good kappa index using reduced amounts of antigen and low conjugate titers, indicating that the test could be efficient for ASFV detection and economical for future commercialization.

**Abstract:**

African Swine Fever (ASF), caused by the African Swine Fever Virus (ASFV), is a highly contagious hemorrhagic disease with high mortality (≈100%) in pigs and is considered the most devastating disease to date. Given the importance of this disease, we aimed to assess the use of the recombinant p30 protein as the sole antigen for the development of an accurate and precise ELISA test (iELISA) for the virus. The recombinant p30 protein (*r*p30) was produced in a bacterial expression system using a SUMO-tagged expression vector. Protein expression was confirmed by Western blot analysis and purified using affinity chromatography. Antigenicity was evaluated in CF-1 mice, which demonstrated the ability to generate high levels of specific antibodies. The *r*p30 showed a sensitivity of 95.6% when used in the development of iELISA, a specificity of 92.3%, and a kappa index (κ) of 0.836. Furthermore, reference sera (OIE-ASF) were used to validate the assays, and the results demonstrated an excellent capacity to detect ASF antibodies using only the *r*p30 antigen up to a serum dilution of 1:100. The inter- and intra-assay variability coefficients were 4.27% and 4.85%, respectively, demonstrating that the assay was accurate and reproducible, allowing its use in seroepidemiological analyses for ASF surveillance.

## 1. Introduction

African Swine Fever (ASF) is an extremely contagious porcine illness marked by hemorrhagic clinical manifestations in production and backyard pigs [1]. Experimental infections in pigs demonstrate that ASF has been characterized as highly infectious, generating clinical signs associated with petechiae, internal hemorrhages, anorexia, reddened skin, bloody diarrhea, respiratory failure, and lethargy [2]. Recently, ASF has become a serious problem in pig farming, and there is currently no safe and effective control method that confers protection to the animals [3]. The first ASF outbreak was reported in Kenya in 1921 [4], and subsequently, it spread rapidly from West Africa to the Iberian Peninsula (Spain and Portugal), where it remained endemic until 1990 [5]. In the last 20 years, the disease has been reported in multiple regions of the European Union, such as Eastern Poland [6]; Georgia in 2007 [7]; Lithuania, with a gradual increase between 2014 and 2017 [8]; and the Baltic region, where the disease spread in 2018, infecting thousands of pigs [9]. Likewise, ASF was detected in Asia, spreading rapidly with an increase in reports in China, Vietnam, and Laos [10], posing a major problem for the swine industry. Recently, in 2021, several outbreaks were confirmed by the United States Department of Agriculture (USDA) in swine production regions in the Dominican Republic and several provinces of Haiti [11,12], representing a serious threat to the Americas.

ASF is induced by the African Swine Fever Virus (ASFV), a double-stranded genome DNA virus belonging to the family Asfarviridae, genus Asfivirus [5,6,7]. At least 25 genotypes have been identified [13], with genotype II being the most widely distributed in Europe, Asia, and the Americas [14]. The genome consists of a linear molecule of about 170–190 kb [8,9], containing 150–167 open reading frames (ORFs) which code for 150–200 proteins, including 68 structural proteins [15,16]. The virus is enveloped and has inner and outer lipid membranes forming an icosahedral capsid [17]. The viral phosphoprotein p30, encoded by the *CP204L* gene, is located in the inner membrane, with a molecular mass of 30 kDa and a length of 194 amino acid residues [15]. The p30 protein is involved in the regulation of transductional functions [16]. It begins before viral DNA synthesis and remains active until the end of the viral cycle [17]. The p30 protein is immunogenic and stimulates the production of antibodies during ASFV infection, which are detected as early as 7 days post-infection in pigs, reaching high levels after 21 days [18]. Serological tests, such as the enzyme-linked immunosorbent assay (ELISA), allow for the early detection of ASFV and hence the formulation of prevention and control strategies for the virus [19]. As a result, the p30 protein has been used for the development of ELISA tests in conjunction with other structural proteins and has shown high sensitivity and specificity against sera from ASF-infected pigs [20,21]. To date, the use of the p30 protein as the sole antigen in the development of a highly sensitive and specific iELISA test shows promise for ASFV detection. In this regard, the aim of this work is to produce the p30 protein recombinantly from the synthetic *CP204L* gene and to develop an iELISA test that would rapidly identify ASF antibodies.

## 2. Materials and Methods

### 2.1. Cloning and Overexpression of ASFV-p30 Protein

The synthetic ASFV-p30 protein gene (CP204L-584 bp) was obtained by end-point PCR from the 2007 Georgia sequence (access number: FR682468) with specific oligonucleotides: 5′-GAGGTCATCTTCAAAACGGAT-3′ (Fw-rp30) and 5′-CTACTAGAGTCTTACCACCTC-3′ (Rev-rp30). These oligonucleotides allowed us to select a 528 bp reading frame to adjust the selected amplicon with the expression vector. The resulting amplicons were ligated into the pASK-IBA33 plus shelterin vector (IBA, St. Louis, Mo, USA) and were restrained in *E. coli* strain TOP10, at the Instituto de Investigación y Tecnología Agroalimentaria, IRTA CReSA, Bellaterra, Spain. The cloned gene was sequenced (chain termination method) and analyzed in the DNASTAR’s Lasergene software version 7.0.0 (DNASTAR, Madison, WI, USA) for the construction of the ASFV-p30 expression plasmid. Subsequent ASFV-p30 subcloning and expression methodologies were carried out using the procedures described by Lara et al. [22]. The Open Reading Frame (ORF) coding for the 528 bp ASFV-p30 protein was introduced into the Champion™ pET SUMO System (Thermo Fisher Scientific, Waltham, MA, USA) and confirmed by orientation analysis. Bacteria prepared with calcium chloride of BL21(DE3) (Invitrogen, Carlsbad, CA, USA) were transformed to overexpress recombinant ASFV-p30 (ASFV-rp30). The overexpression and purification of ASFV-*r*p30 were carried out in 100 mL of LB medium inoculated with a pre-culture of strain BL21-ASFV-*r*p30 (10 mL of LB plus kanamycin, 50 µg/mL) starting at 0.1 optical densities at 600 nm (OD_600nm_). The inoculated medium was incubated at 37 °C at 250 rpm until 0.5 OD_600nm_ was reached. Subsequently, the inducer (Isopropyl-beta-d-thiogalactopyranoside) was inoculated as an inducing agent at 1.50 mMol. The medium was grown to 16 h under the same conditions mentioned above. After this time, the inclusion bodies (ICs) were removed from the cells by mechanical disruption using a homogenizer and dissolved in N-laurylsarcosine (5%) in 50 mM Tris-HCL (pH 8). With the solubilized ICs, the ASFV-*r*p30 protein was purified via immobilized metal ion affinity chromatography (IMAC) using a HisTrap^®^ Ni-NTA agarose column (GE Healthcare, Chicago, IL, USA). To assess protein purification, the elution fraction was analyzed on acrylamide gel and protein immunoblotting (WB) using anti-6His (Invotrogen, Rockford, IL, USA), in addition to the Bradford method for quantification of the purified protein.

### 2.2. Antigenic Evaluation of ASFV-rp30 Protein In Vivo

To determine the antigenic capacity of ASFV-*r*p30, 24 three-week-old female mice of the Crl:CF-1 strain were divided into three groups (n = 8). The mice were inoculated subcutaneously into the skin folds of the neck with a dose of 200 µL of 5 µg total pure protein in PBS as the vehicle. The immunization schedule was as follows: group 1—PBS (blank control); group 2—ASFV-*r*p30 plus PBS (positive control); group 3—ASFV-*r*p30 plus Matrix-M^TM^ adjuvant (Isconova AB, Uppsala, Sweden). The mice were immunized twice 14 days apart and were bled in the tail vein every 7 days until day 35 post-inoculation. For antibody determination, the iELISA was performed based on previous experiments with some modifications [23]. ASFV-*r*p30 was used as an antigen, with 50 ng per well of a 96-well plate. The results were statistically compared by bleeding days using a two-way ANOVA test, and the protein-alone group was compared with the protein-plus-adjuvant group. Statistically significant differences were considered with a 95% confidence interval (* *p* < 0.05; ** *p* < 0.005; *** *p* < 0.0005). Graphs were generated and statistical analyses were performed in SigmaPlot 12.5 software (Systat Software Inc., San Jose, CA, USA) and NCSS 20.0.8 software (Kaysville, UT, USA). The procedures were performed on the murine models following NOM-062-ZOO-1999, SAGARPA, Mexico, according to National Research Council (NRC) [24]. The animal assays were authorized by CBCURAE-2017/001, CENID-Salud Animal e Inocuidad/INIFAP.

### 2.3. Optimization of ASFV-rp30 Coated Indirect ELISA

The conditions for iELISA standardization were determined by assessing the best concentrations of antigen and sera. Pure ASFV-*r*p30 was used at a concentration of 50–500 ng in all plate using Carb-Bicarb buffer (50 mM), pH 9.6, in 96-well MaxiSorpTM plates (Nalge Nunc International Corp., Rochester, NY, USA), with 100 µL in each well. The plates have been incubated at 4 °C overnight, followed by washing with phosphate buffer-Tween 0.1% solution, and blocked with 7% skim milk. at 37 °C for 1.5 h. ASFV-positive sera from pigs experimentally infected with ASFV strain NHV/68 and negative sera were diluted (1:50 to 1:200) in PBS-Tween, plated on the antigen-treated plates at a volume of 100 µL per well, and subsequently incubated at 37 °C for 1 h. Then, the plates were washed 4 times with PBS-Tween, and the secondary antibody, anti-pork IgG/HRP (Sigma, St. Louis, MI, USA), has been diluted in 1:15,000 and 1:20,000 dilutions at a volume of 100 µL per well. The plate was again incubated at 37 °C for 1 h. Finally, the plates were washed 5 times, and 100 µL of tetramethylbenzidine chromogenic-solution (SeraCare, Milford, MA, USA) was placed in all plates and left to react for approximately 15 min at room temperature (25–28 °C) in complete darkness. The reaction was stopped by adding 100 µL of the stop solution, 2 N sulfuric acid. Finally, the absorbance of the plate wells was read at a wavelength of 450 nm (OD_450nm_).

It was run 10 times with samples from pigs strongly positive for ASF to determine the coefficient of variation (*CV*). For validation of the tests, ASF-positive (strain NHV/68) (n = 24) and negative (n = 25) serums were evaluated. All reference and these serums had previous been evaluated using the established immunoperoxidase test (IPT) and blocking-ELISA for ASFV antibody detection kit (Ingenasa-Ingezim PPA Compac K3; Ingenasa, Madrid, Spain), according to OIE validation guidelines [25], using a validation index of 1:1280 as strongly positive (C++) and 1:180 as weakly positive (C+). The sera used correspond to a collection from an outbreak of ASFV that occurred in Spain in the 1990s and experimental infected pigs with ASFV (NHV/68 strain); ASF-negative controls were also included in the assays (C−). The results of the iELISAs allowed us to calculate the coefficient of variation (*CV*), i.e., an inter-assay *CV*, average of strong (C++) and negative (C−) sera of plates evaluated; a value less than 15% was considered acceptable. This was calculated as follows [26,27]:

%CVinter assay=CV of strong positive controls+CV of negative controls2

In another way, for Intra-assay precision, each serum sample was evaluated in duplicate. The *CV* percentage was calculated for each sample evaluated, obtaining the standard deviation of both results, divided by the mean and multiplied by 100. The average of the individual CVs was reported as an intra-assay CV, and those presenting less than 10% reflected good assay performance. The calculation was as follows:

$$\%{CV}_{intra\;assay}=\bar{\%CV}$$

### 2.4. Statistical Analysis

For different analyses, the Win Episcope 2.0 program [28] was used (α = 0.05). Sensitivity and specificity were obtained by comparing the true positive and negative sera, considering the data obtained by IPT, using a 2 × 2 contingency table. The percentage of sensitivity and specificity was calculated as follows:

$$\%Sensitivity=\left[\frac{a}{a+c}\right]\times 100$$

$$\%Specificity=\left[\frac{d}{b+d}\right]\times 100$$

where *a* = total of true positives, *b* = total of false positives, *c* = total of false negatives, and *d* = total of true negatives.

Two analyses were validated using the Cohen’s kappa coefficient (κ), using the IPT assay as the reference test and was calculated as follows:

$$K=\frac{\left(Po-Pe\right)}{\left(1-Pe\right)}$$

where

$$Po=\frac{a+b}{N}$$

$$Pe=\frac{rt+su}{N\times N}$$

r=a+bt=a+cs=c+du=b+dN=a+b+c+d

Cohen’s kappa coefficient was interpreted as follows: 0.81 as excellent determination, 0.61 to 0.80 as substantially perfect determination, 0.41 to 0.60 as good determination, 0.21 to 0.40 as moderate determination, 0.01 to 0.20 as poor determination, and 0.00 as non-determinant [29,30].

### 2.5. Indirect ELISA Cut-Off Determination

The cut-off was determined using the serum antibody levels allowed for distinguishing between sera from positive and negative animals [31]. For determination, control sera confirmed as true positive (n = 22) and true negative (n = 24) by IPT were evaluated to standardize the test. The mean value of the negative sera at OD_450nm_, added a pair of standard deviations (SDs), was established as the cut-off, with a last critical score of $\bar{x}$ + 3 SD. Values above the critical point were considered positive for ASF. Values between the critical point and the cut-off point were deemed suspicious, while values below the cut-off point were regarded as negative.

## 3. Results

### 3.1. Development and Expression of the Recombinant System for rp30

**Figure 1:**
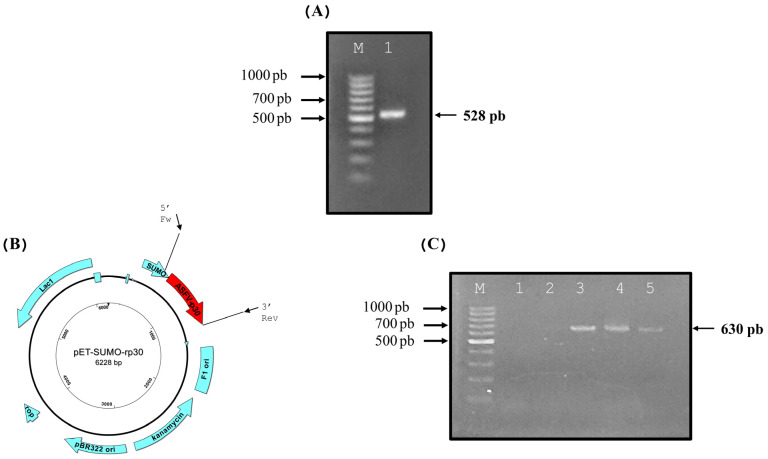
(**A**) Amplification of the *CP204L* gene, with a size of 528 bp (M—marker; 1—amplicon *CP204L*). (**B**) Expression vector pET-SUMO-*r*p30, with the insert corresponding to the *CP204L* gene (ASFV-p30) and primers at the corresponding positions for targeting analysis. (**C**) Targeting analysis of the *CP204L* gene in the expression vector (M—marker; 1/2—blank control; 3/5—samples of the expression vector with the insert).

The full-length reading frame *CP204L*, which encodes the 528 bp p30 protein, was amplified using specific oligos at the expected molecular weight with no nonspecific products (Figure 1A) and then successfully subcloned into the expression vector pET-SUMO, as shown in the representative expression vector schematic (Figure 1B). The *CP204L* gene, 528 bp in length when ligated into the expression vector, allows the pET-SUMO-*r*p30 system to be obtained, which can be verified by orientation analysis using two specific primers: SUMO-Fw, which hybridizes in a region of the expression vector coding for the SUMO protein, and *ASFV*-p30 3′ Rev, which hybridizes at the 3′ end of the insert and whose amplification product increases by approximately 102 additional bp (630 bp) (Figure 1C).

**Figure 2:**
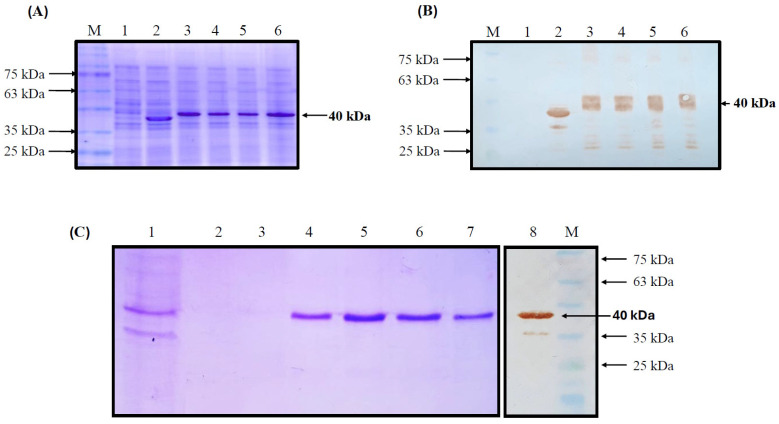
Analysis of *r*p30 protein expression in the BL21/DE3 expression system by SDS-PAGE (**A**). Protein specificity was determined by Western blot (**B**) using 6X His-tag antibody (M—marker; 1—negative [Untransformed BL21/DE3]; 2—positive [BL21—CAT]; 3/6—BL21-ASFV-p30). SDS-PAGE and Western blot analysis of IMAC-purified *r*p30 protein (**C**) (1—CI; 2—unbound; 3—washed; 4/7—specific expression bands of the *r*p30 protein in IMAC elutions).

The recombinant pET-SUMO-*r*p30 system efficiently transformed the production strain *E. coli* BL21/DE3 and was induced to produce *r*p30 by IPTG. The recombinant protein was successfully expressed in the expression strain, confirmed by the presence of an overexpressed band at the expected molecular weight of 40 kDa on SDS-PAGE in various transformed strains. Such a band was not observed in the negative control strain (untransformed BL21/DE3) or in the positive control strain (overexpressing a 37 kDa recombinant protein) (Figure 2A). Western blot analysis using 6×-his-tag antibody followed by HRP-conjugated secondary antibody for visualization confirmed the presence of a specific signal for these overexpression bands in the analyzed strains and the positive control (37 kDa recombinant protein), confirming the recovery of *r*p30 (Figure 2B). The *r*p30 protein was recovered from the cells in insoluble form as inclusion bodies, which were dissolved in sarcosyl and subsequently purified by IMAC. Figure 2C shows the results of purification with SDS-PAGE and the Western blot analysis of the recombinant, which was identified as a single band with a specific signal at 40 kDa in the elution samples obtained by chromatography, without the presence of contaminating protein bands. The final protein concentration was 30 µg/mL, indicating a production yield of 3 mg per 100 mL.

### 3.2. Humoral Evaluation of rp30

**Figure 3:**
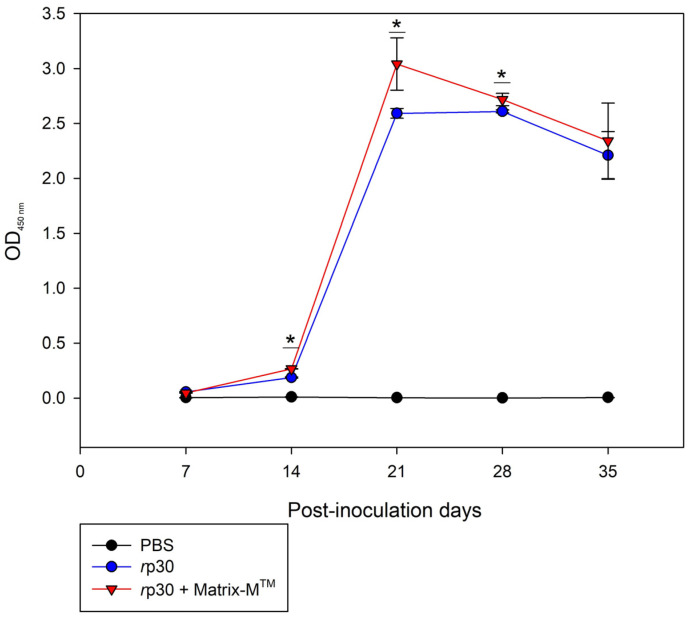
Humoral evaluation of *r*p30 in CF-1 strain mice. * Statistically significant differences were considered when *p* < 0.05 between group 2 (*r*p30) and group 3 (*r*p30 + Matrix-M^TM^).

The antibody production capacity of *r*p30 in CF-1 mice is represented as the production kinetics, obtained from the data provided by the iELISA, using the pure protein as the antigen. Figure 3 shows the antibody production in mice, and it was observed that the group inoculated with *r*p30 alone, 14 days post-inoculation (2nd dose), produced high levels of specific antibodies, with a significant difference (* *p* ≥ 0.05) on day 21 compared to the group administered with the adjuvant. However, a similar effect to the group inoculated with rp30 plus adjuvant was observed throughout the other days, indicating that the protein alone has a high antigenic effect, which was maintained until day 35. These results imply that the *r*p30 protein produced through the bacterial expression system contains the necessary elements to generate specific antibodies and is capable of sustaining this production over time.

### 3.3. Standardization and Validation of the iELISA Using rp30 as the Antigen

**Figure 4:**
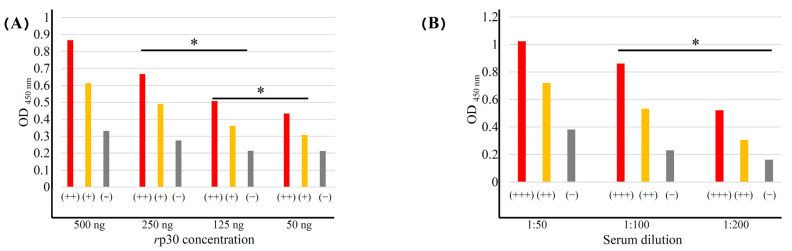
Evaluation of the optimal concentration of antigen (**A**) and serum (**B**) for iELISA standardization. Optimal conditions were considered when 50 ng was used for each plate and 1:100 dilutions were used for the sera evaluated. Statistically significant differences were considered at * *p* ≤ 0.05.

The optimal conditions used for iELISA development corresponded to the block titration model; it was determined that 50 ng of protein with CC buffer was sufficient to antigenize each well of the 96-well plates and efficiently recognize the antibodies. A dilution of 1:100 was most suitable for serum evaluation in the iELISA with an incubation period of 1 h at 37 °C. The optimal dilution of anti-pig HRP conjugate used in the reference sera was 1:20,000. The optimal reaction or incubation time when TMB was incorporated was 15 min (Figure 4). For assessing the repeatability of this assay, 10 runs were completed using HRP reference sera (positives and negatives). The frequency of distribution of positive and negative sera was compared with previous evaluations [32], obtaining an inter-assay CV of 4.27%, which indicates the stability and consistency of iELISA as well as its high reproducibility, and an intra-assay CV of 4.85%, reflecting good assay performance.

### 3.4. Cut-Off Point, Sensitivity, Specificity, and Kappa Index of the iELISA

**Figure 5:**
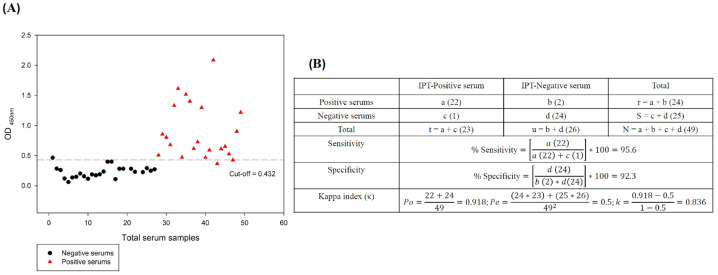
Serological distribution of positive and negative reference sera evaluated in iELISA with *r*p30 (**A**), with a cut-off point of 0.432. The 2 × 2 contingency table (**B**) for the analysis of 49 sera (24 positive and 25 negative) to determine sensitivity, specificity and κ.

The cut-off was determined by evaluating 25 negative reference sera under the optimized iELISA criteria, resulting in 0.232 at a SD of 0.1. Cut-off score plus 2 SD was 0.432 (Figure 5A). Samples having an OD greater than the cut-off were judged positive, while the ones that remained below the cut-off were declared negative. ODs that remained between the critical point and the cut-off point were deemed suspicious, requiring confirmation using the IPT.

**Figure 6:**
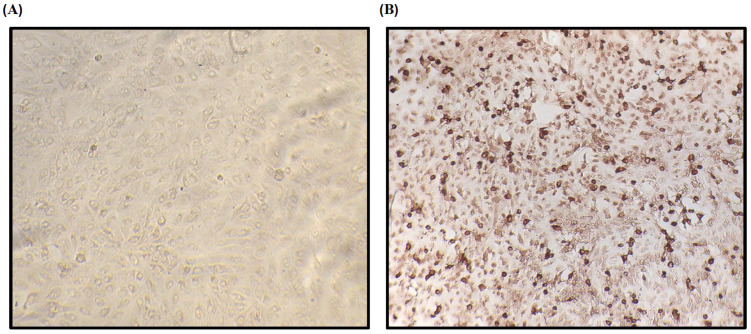
Indirect immunoperoxidase test (IPT) as a confirmatory test for ASF-negative (**A**) and -positive (**B**) serum samples.

Sensitivity, specificity, and the κ index were evaluated using a 2 × 2 contingency table. Under the experimental conditions mentioned above and using the reference serum bank of the OIE-ASF reference laboratory, sensitivity and specificity were 95.6% and 92.3%, respectively. Κ value has been 0.836, which indicates a perfect correlation between the iELISA evaluations and the IPT reference test (Figure 5B). In the IPT, considered the standard and reference test for ASF diagnosis in Spain, a specific intracellular signal was observed for ASFV-infected cells corresponding to cells containing active virus replication and thus recognized by the antibodies present in the sera of infected pigs, contrary to the negative sera samples, in which these antibodies were not observed in the cells (Figure 6). Finally, all the sera used in this study correspond to the expected result, which confirms the specificity of the developed immunoassay.

## 4. Discussion

African Swine Fever is an infectious disease caused by ASFV that affects pigs and has been found to cause 100% mortality in production and backyard farms, generating considerable productive and economic losses in the affected countries [33]. Genotype II ASFV has a wider distribution in countries with high pig production, such as China and Russia as well as other European and Asian countries [34]. Considering the impact of the disease and that genotype II strains present a greater threat owing to their wide distribution, this study aimed to develop a recombinant p30 protein from the ASFV/Georgia/2007 sequence and to implement, evaluate, and validate a sensitive and specific iELISA. ELISA tests facilitate the early detection of infectious agents, and their development is implemented with follow-up validation and standardization, allowing for their effective use [35]. Thus, the *r*p30 iELISA would enable the identification of acutely infected and convalescent animals, maintaining surveillance and early detection of ASFV. In North and Central America, although there have been no reports of ASFV, there is a surveillance alert for its monitoring; therefore, the development of biological strategies for the prevention and control of ASF is paramount [35]. However, because most commercial ELISA kits are located outside these countries, their high cost would reduce the possibility of their timely acquisition and application, especially in Latin America.

The ASFV p30 protein presents highly immunogenic regions in its amino acid residue sequence, particularly highly conserved among genotype II [18], which makes it a potential candidate for the development of biological tools against ASF. Thus, we assume the p30 protein is a valuable diagnostic tool for detecting ASFV, our newly developed iELISA assay could be used for the universal detections of genotype II (100% homology) and sequences from genotype I isolates (97.5% homology); yet, evaluations are needed to determine its practical applicability in health surveillance. Therefore, the sequence of the *CP204L* gene, which encodes the p30 protein of the ASF/Georgia/2007 strain, was selected and amplified, ensuring that the recombinant protein produced could be used as the sole antigen in the development of an iELISA.

The recombinant protein was developed in an accessible, economical, and high-quality prokaryotic expression system using the pET-SUMO expression vector, which has shown high versatility in recombinant protein production using *E. coli* expression systems. However, despite not generating post-translational modifications, the conservation of epitopes in peptides allows their safe and effective use [36,37]. The *r*p30 protein was efficiently overproduced in the prokaryotic expression system in the form of CI and was detected via Coomassie and Western blot analysis with a specific signal at approximately 40 kDa due to the SUMO and 6-His tags added by the expression vector. Subsequently, it was purified in a single step by IMAC, and Bradford quantification determined that the expression system could generate sufficient quantities to carry out subsequent evaluations.

The pET-SUMO technology allowed for the efficient production of the recombinant protein and ensured its quality as a viral antigen with antigenic and immunogenic properties. These properties were not reduced by the incorporation of the 6-His and SUMO tags, which confer advantages regarding the solubility and stability of the recombinant protein [38]. In contrast, the evaluation of the humoral response corroborated the antigenic and immunogenic capacity of *r*p30, demonstrating that the protein retains characteristics similar to the native one and, alone or with an adjuvant, can generate high levels of antibodies, which were detected from day 14 post-immunization. The highest peaks occurred from day 21, corresponding to 7 days after the second immunization, indicating that few doses were needed to generate a strong and long-lasting humoral response, which was maintained in mice for up to 35 days. These results highlight the possibility of building an immunization model using the *r*p30 protein as an antigen, which, when coupled with a lipid adjuvant, would allow for better distribution and uptake by antigen-presenting cells, thereby increasing the immune response [39].

Obtaining *r*p30 allowed for the development of an iELISA for detecting antibodies against ASFV in infected animals. The developed test showed good sensitivity and specificity, allowing for comparison with commercial tests that use p30 in combination with other antigens. For example, the commercial Svanova-iELISA, which uses the recombinant p30 protein (Svanovir ASFV-Ab; Boehringer Ingelheim Svanova, Uppsala, Sweden), has a specificity of 91.4%; however, a high number of false positives have been reported. In contrast, a multi-antigen iELISA test (IDvet-ELISA), which detects antibodies against others proteins of ASFV and has a specificity of 100% [40]. Other non-commercial developments of iELISA tests that use the recombinant p30 protein differ from our work because of the expression system used to produce the recombinant protein and the optimal conditions for the iELISA. For example, an iELISA that used p30 as an antigen, obtained from cell culture (HEK293F line), evaluated 90 sera negative for ASFV, obtaining a cut-off value of 0.337, lower than the cut-off value of the ASFV Detection ELISA kit (PT Biotek) of 0.5 [41]. In this study, a cut-off point of 0.432 was considered due to the importance and rapid spread of the disease, so samples with values between the critical point and the cut-off point were considered suspicious and must be evaluated with a confirmatory test, such as the IPT, demonstrating the feasibility of using the recombinant protein obtained from the prokaryotic system in the development of rapid detection systems for ASF. However, developments based on animal cells capable of carrying out post-translational modifications continue to have higher production and maintenance costs than bacterial systems. An indirect ELISA using dual p22 and p30 proteins obtained significant results using up to 400 ng of the antigen [42]. Our results show that using 50 ng of the *r*p30 protein yields high sensitivity, indicating that our development would allow for reduced manufacturing costs. Similarly, another immunodetection system using a chimeric protein with 500 ng of antigen and a serum dilution of 1:40, obtained a sensitivity value of 97.5% and a specificity value of 95.4% [43]. Our assays showed a sensitivity value of 95.6% and a specificity value of 92.3% using 50 ng of *r*p30 antigen with a serum dilution of 1:100, highlighting the recognition capacity of our recombinant antigen. The p30 protein has been targeted for the development of highly sensitive tests, such as the one reported by Liu et al. in 2021 [44]. However, because the disease affects both developed and developing countries, as in most countries in the Americas, the availability of sensitive and affordable tests is imperative, allowing for greater reach, particularly in farming areas with limited opportunities for ASF detection.

## 5. Conclusions

African Swine Fever is a highly contagious disease with a high mortality rate in pigs, posing a global economic risk. Due to its rapid spread among countries with high pig production, there is an urgent need to develop effective and accessible tools for developed and developing countries, such as those in the Americas that could be used in biosecurity laboratories at level 2 (BSL 2). These tools would allow for the detection of antibodies in animals or samples, which would be beneficial for the detection of ASF in imported animals or those at risk from ASFV transmission vectors.

According to our results, the *r*p30 protein was successfully produced in the prokaryotic system, which, in addition to exhibiting good production yields, exhibited antigenic characteristics capable of generating a strong humoral response. The developed iELISA is a biotechnological tool capable of recognizing the presence of antibodies against ASFV in disease-free sites in a timely manner, given its high sensitivity and specificity for the recombinant antigen.

## Data Availability

The original contributions presented in this study are included in the article/Supplementary Materials. Further inquiries can be directed to the corresponding author.

## Supplementary Materials

The following supporting information can be downloaded at: https://www.mdpi.com/article/10.3390/vetsci12100995/s1, Figure S1: Original image of Figure 1A; Figure S2: Original image of Figure 1C; Figure S3: Original image of Figure 2A; Figure S4: Original image of Figure 2B; Figure S5: Original image of Figure 2C.
