# Supplementary material for: Development and Standardization of Indirect ELISA for African Swine Fever Virus Using Recombinant p30 Protein Produced in Prokaryotic System
Source: Vet Sci. 2025 Oct 15;12(10):995. doi: 10.3390/vetsci12100995 (PMC12568178; doi:10.3390/vetsci12100995)

Figure S1: Original image of Figure 1 A

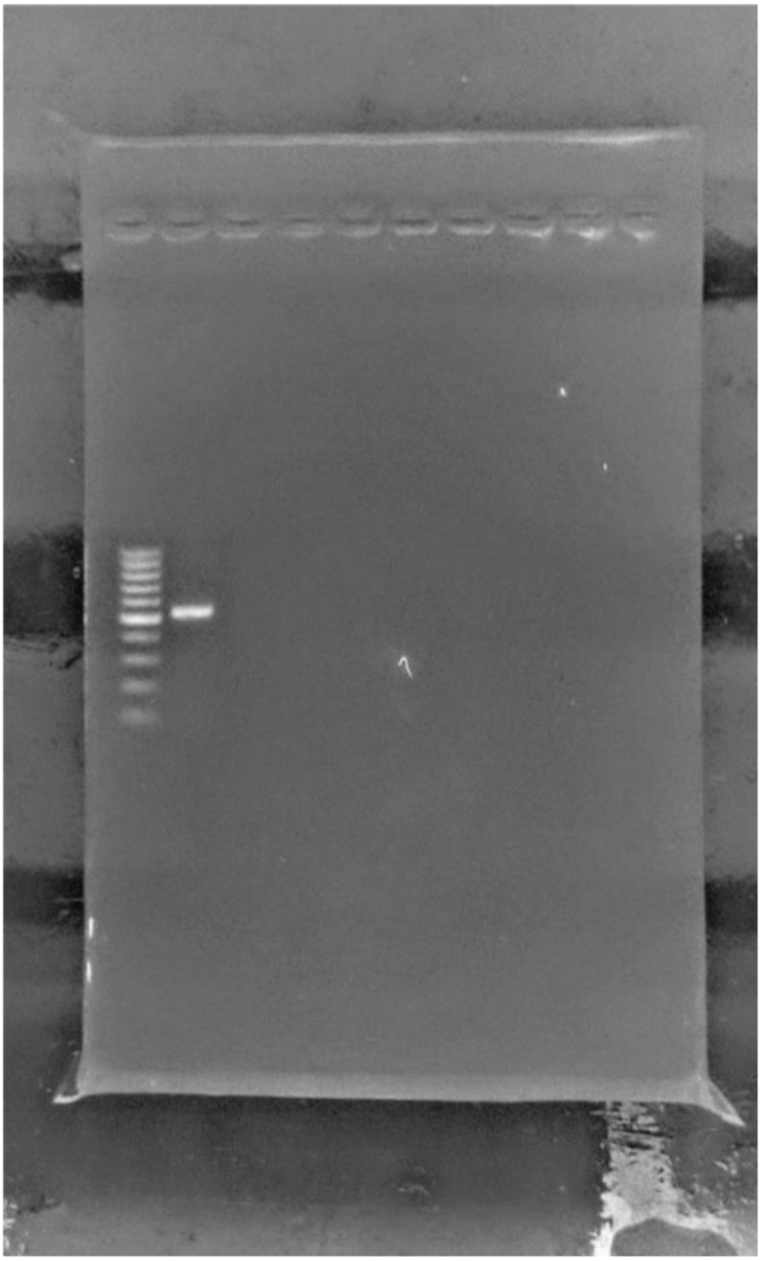

Figure S2: Original image of Figure 1 C

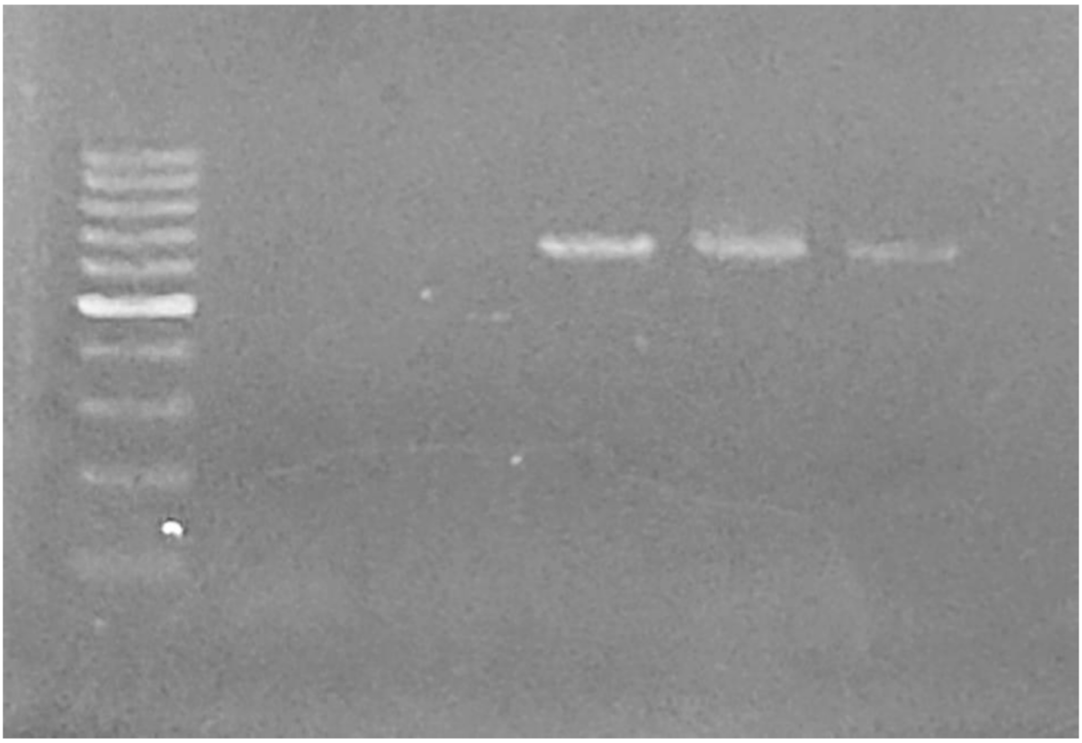

Figure S3: Original image of Figure 2 A

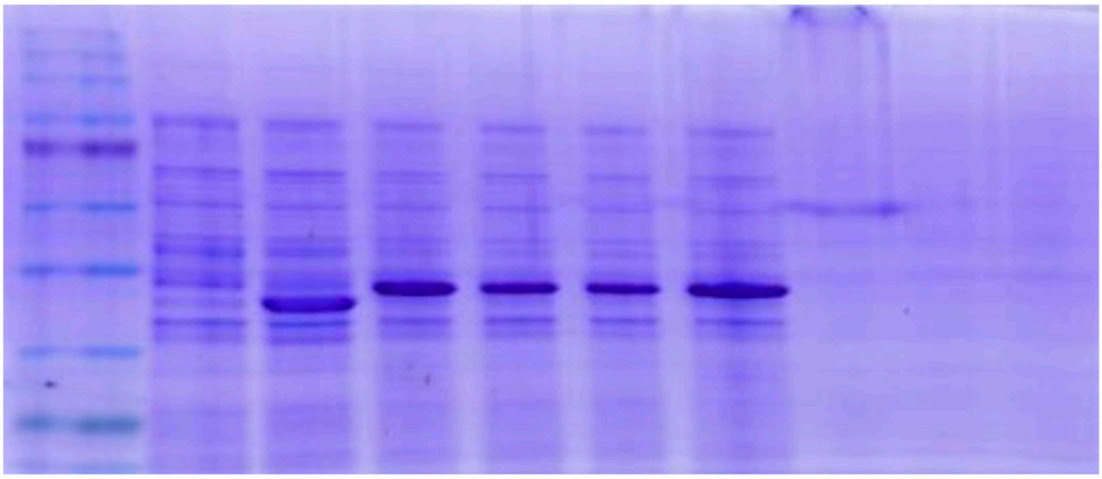

Figure S4: Original image of Figure 2 B

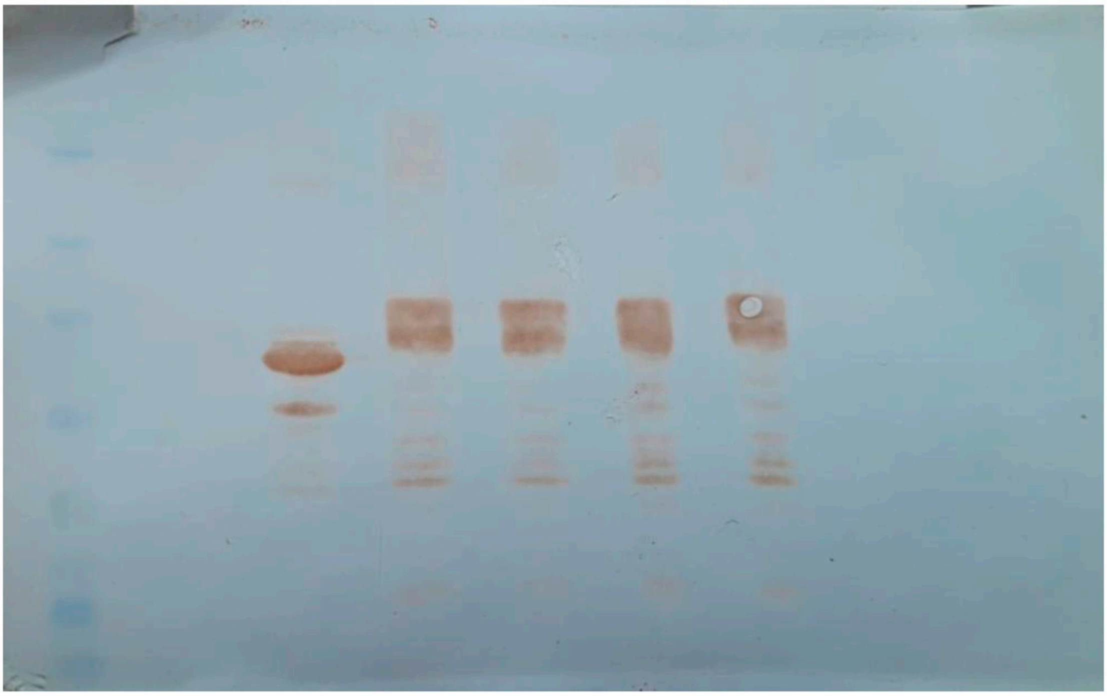

Figure S5: Original image of Figure 2 C

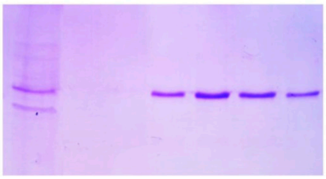

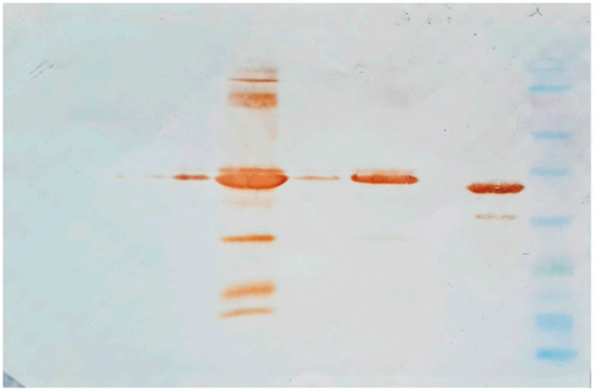

Supplement: Supplementary file 1 [file vetsci-12-00995-s001.zip › vetsci-3909071-supplementary.pdf]
